# Supplementary material for: Mucosal Vaccination Against SARS-CoV-2 Using Human Probiotic Bacillus subtilis Spores as an Adjuvant Induces Potent Systemic and Mucosal Immunity
Source: Vaccines (Basel). 2025 Jul 21;13(7):772. doi: 10.3390/vaccines13070772 (PMC12298100; doi:10.3390/vaccines13070772)
Supplement: Supplementary file 1 [file vaccines-13-00772-s001.zip › vaccines-3694116-supplementary.pdf]

Table S1. Reagents, fluorophores, clone numbers, product codes, and dilutions used for flow cytometry. All the reagents were supplied by BioLegend.

Reagent	Fluorophore	Clone	Product code	Dilution
Anti-mouse/human CD45R/B220	Alexa Fluor® 700	RA3-6B2	103231	1:200
Anti-mouse CD45	Alexa Fluor® 700	30-F11	103127	1:200
Biotin anti-mouse IgA	N/A	RMA-1	407003	1:200
Streptavidin	APC	N/A	405207	1:200
Anti-mouse CD103	APC	2E7	121413	1:200
Anti-mouse CD138	Brilliant Violet 421™	281-2	142507	1:100
Anti-mouse CD8a	Brilliant Violet 510™	53-6.7	100751	1:200
Anti-mouse IgD	Brilliant Violet 605™	11-26c.2a	405727	1:200
Anti-mouse CD19	Brilliant Violet 650™	6D5	115541	1:200
Anti-mouse/human CD44	Brilliant Violet 650™	IM7	103049	1:200
Anti-mouse CD69	Brilliant Violet 785™	H1.2F3	104543	1:200
Anti-mouse CD3	FITC	17A2	100203	1:500
Anti-mouse CD4	Pacific Blue™	RM4-5	100534	1:200
Anti-mouse CD38	PE	90	102707	1:200
Anti-mouse IL-4	PE	11B11	504103	1:200
Anti-mouse IFN-γ	PE/Cyanine7	XMG1.2	505825	1:200
Goat anti-mouse IgG	PE/Cyanine7	Poly4053	405315	1:200
Anti-mouse IL-17A	PE/Dazzle™ 594	TC11-18H10.1	506937	1:50
Anti-mouse CD3	PerCP/Cyanine5.5	17A2	100217	1:200

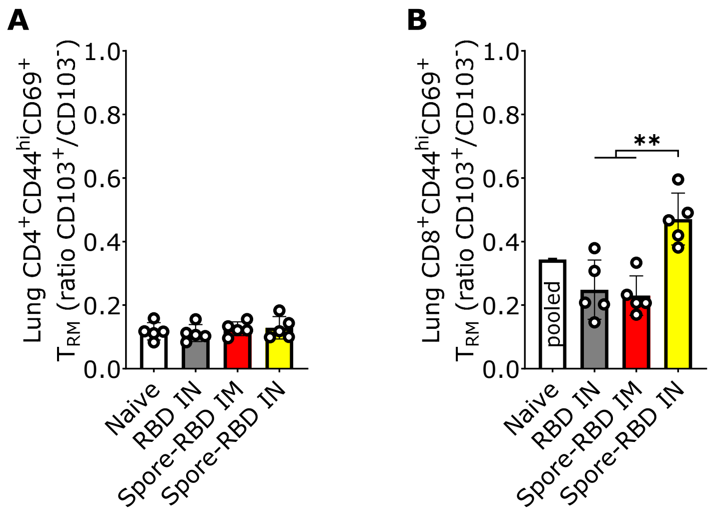

Figure S1. CD103⁺/CD103⁻ ratios of CD4⁺ and CD8⁺ T_{RM} after SARS-CoV-2 RBD intranasal immunization adjuvanted with *Bacillus subtilis* DG101 spores. (**A**,**B**) $CD103^+/CD103^-$ ratios of $CD4^+$ (A) and $CD8^+$ T_{RM} (B) in the lungs of naive mice, or mice immunized with RBD alone intranasally (RBD IN), or with RBD adsorbed onto *B. subtilis* DG101 spores either intramuscularly (spore-RBD IM) or intranasally (spore-RBD IN). All data are depicted as mean ± SD, except naive mice (controls) in (B), which are shown as the ratio of the pooled data. Each experimental group consists of 5 animals, and each dot represents an individual

animal. Statistical significance was calculated by one-way analysis of variance (ANOVA); ** $p < 0.01$.
